# Mean Platelet Volume, Vitamin D and C Reactive Protein Levels in Normal Weight Children with Primary Snoring and Obstructive Sleep Apnea Syndrome

**DOI:** 10.1371/journal.pone.0152497

**Published:** 2016-04-07

**Authors:** Anna Maria Zicari, Francesca Occasi, Federica Di Mauro, Valeria Lollobrigida, Marco Di Fraia, Vincenzo Savastano, Lorenzo Loffredo, Francesco Nicita, Alberto Spalice, Marzia Duse

**Affiliations:** 1 Department of Pediatrics, Sapienza University of Rome, Rome, Italy; 2 Department of Internal Medicine and Medical Specialties, Sapienza University of Rome, Rome, Italy; Charité - Universitätsmedizin Berlin, GERMANY

## Abstract

**Introduction:**

Studies on Mean Platelet Volume (MPV) in children with Sleep Disordered Breathing (SDB) report conflicting results and the hypothesis of an intermittent hypoxemia leading to a systemic inflammation is reaching consensus. Vitamin D exerts anti-inflammatory properties and its deficiency has been supposed to play a role in sleep disorders. Emerging interest is rising about Primary Snoring (PS) since it is reasonable that also undetectable alteration of hypoxia might predispose to an increased production of inflammatory mediators. In this perspective, in a group of children affected by SDB, our aim was to investigate MPV, vitamin D and C Reactive Protein (CRP) levels, which had been previously evaluated separately in different studies focused only on Obstructive Sleep Apnea Syndrome (OSAS).

**Materials and Methods:**

We enrolled 137 children: 70 healthy controls (HC), 67 affected by SDB undergoing a polysomnographic evaluation, 22 with a diagnosis of PS and 45 with a diagnosis of OSAS. All patients underwent routine biochemical evaluations including blood cell counts, CRP and vitamin D.

**Results:**

Children affected by SDB had a mean age of 8.49±2.19 and were prevalently males (23 females, 34%; 44 males, 66%). MPV levels were higher in OSAS and PS when compared to HC; platelet count (PLT) and CRP levels were higher while Vitamin D levels were lower in children with SDB when compared to HC. MPV levels were correlated with PLT (r = -0.54; p<0.001), vitamin D (r = -0.39; p<0.001) and CRP (r = 0.21; p<0.01). A multiple regression was run to predict MPV levels from vitamin D, CRP and PLT and these variables significantly predicted MPV (F = 17.42, p<0.0001; adjusted R2 = 0.37). Only platelet count and vitamin D added statistically significantly to the prediction (p<0.05).

**Conclusion:**

The present study provides evidence of higher MPV and lower vitamin D levels in children with PS as well as in children with OSAS, and supports the underlying inflammation, hence, highlighting the importance of an early diagnosis of this previously considered benign form of SDB.

## Introduction

Sleep disordered breathing (SDB) is very common in children [1–3] and includes diseases ranging from Primary Snoring (PS) to Obstructive Sleep Apnea Syndrome (OSAS).[4] Children affected by OSAS have intermittent episodes of complete or partial obstruction leading to intermittent desaturations and/or arousals, and this condition of intermittent hypoxemia (IH) and reoxygenation damages the endothelium,[5] determining significant changes in the cardiovascular system.[6] Platelet size, measured by Mean Platelet Volume (MPV), is a potential marker of platelet reactivity since larger platelets are metabolically and enzymatically more active showing a greater protrombotic potential.[7] In this perspective, it may be regarded as a potentially useful biomarker of platelet activity in the setting of cardiovascular disease.[8] Of note, platelet activation plays a fundamental role in the pathophysiology of thrombosis and other inflammatory diseases, and MPV is influenced by thrombopoietin and numerous inflammatory cytokines regulating thrombopoiesis, hence it is a reflection of both proinflammatory and prothrombotic conditions.[9]

The association between increasing MPV and disease severity has been demonstrated in adults [10] and has been the object of many studies in children with OSAS. One recent study highlighted that MPV is higher in patients with severe obstructive sleep apnea than in healthy patients [11] and Sagit et al.[12] showed that MPV is increased in patients with septal deviation and snoring due to severe upper aiway obstruction. In this complex relation beetween SDB and MPV, SDB-related hypoxemia and/or sleep fragmentation may lead to a systemic inflammation as supported by the higher C reactive protein (CRP) levels in children with SDB,[13] in particular when a neurocognitive dysfunction has developed.[14] In this perspective, another potential interesting factor might be represented by vitamin D which may control the release of proinflammatory cytokines and its deficiency has been associated with higher CRP [15] and MPV.[16]

Although, in subjects affected by PS, OSAS cannot be diagnosed according to international polysomnographic parameters, it is reasonable that also mild episodes of IH might predispose to cell stress and determine an increased production of inflammatory mediators. In this perspective, current assessment techniques might not be sensitive enough to detect subtle gas exchange abnormalities or sleep disturbance in this setting,[17] since many studies demonstrated that children with PS, just like patients affected by OSAS, have cognitive and behavioral morbidity, increased blood pressure and reduced arterial distensibility.[18,19] Recently, our research group have demonstrated that children with PS have significantly higher levels of resistin compared to control group,[20] and that NOX2-derived oxidative stress is involved in artery dysfunction in SDB children, including both PS and OSAS.[21]

In this perspective, our aim was to investigate, also in children with PS, MPV, vitamin D and CRP levels which had been previously evaluated separately in different studies focused only on OSAS.

## Materials and Methods

We enrolled 67 consecutive SDB children and 70 healthy controls (HC) recruited between February 2014 and June 2015 at the Pediatrics Department of “Sapienza” University of Rome. We performed, on each subject, a complete physical examination, a full medical history, polysomnography and venous blood sampling. Snoring was investigated by a validated SDB questionnaire including questions about snoring frequency per week.[22,23] SDB children were divided in PS (n = 45) and OSA (n = 22). PS was defined as snoring without apnea, sleep abnormalities or gas exchange abnormalities.[24] According to the recent guidelines,[24] OSA is a breathing disorder characterized by prolonged partial upper airway obstruction and/or intermittent complete obstruction during sleep, with loss of normal ventilation during sleep and normal sleep patterns. HC were consecutively selected during a routine health check-up and they did not report symptoms of habitual snoring and/or rhinitis.

Exclusion criteria were as follows: presence of epilepsy, acute or chronic cardio-respiratory diseases, neuromuscular diseases, acute or chronic inflammatory diseases, liver disease, serious kidney disorders, overweight.

The study was approved by the Ethical Committee of “Sapienza University of Rome” and written parental informed consent was obtained.

### Adenoids and tonsils

Nasal Fibroptic Endoscopy was executed by an expert otorhinolaryngologist using a 2.7 mm diameter endoscope. Adenoids dimensions were graded using criteria previously described by Cassano et al.[25]: grade 1 for adenoids occluding more than 25% of the choanal opening, grade 2 for adenoid tissue limited to the upper half (<50%), grade 3 for adenoid tissue extending over the rhinopharynx (<75%) with obstruction of choanal opening and partial closure of tube ostium, grade 4 for almost total obstruction. Moreover, tonsillar hypertrophy was classified as 1 when tonsils were observed in the tonsillar pillar, 2 when they protruded out of the tonsillar pillar, 3 when they reached the space between anterior tonsillar pillar and uvula, 4 when they attached to the uvula.[26]

### Polysomnography

Standard overnight polysomnography recordings were performed by a Grass Heritage polygraph as previously described.[2] The following variables were recorded: body position and oxygen saturation, thoracic and abdominal respiratory effort, six electroencephalogram channels, right and left electrooculogram, electromyogram of left and right tibialis anterior muscles, chin electromyogram, electrocardiogram nasal flow.[2] Obstructive apnea was defined as absence of airflow with constant respiratory effort for more than two baseline breaths, independently of arterial oxygen saturation changes.[2] Hypopnea was defined as a reduction of at least 50% but not more than 90% in the amplitude of the airflow signal and it was only quantified if longer than two baseline breaths and associated with arousals and/or an oxygen desaturations >3%.[27] The obstructive apnea-hypopnea index (OAHI) was defined as the total number of apneic and hypopneic episodes per hour of sleep.[27] PS was diagnosed when OAHI was <1 and SpO2 nadir ≥90%; OSA was diagnosed if OAHI was ≥1.[28,29]

### Blood sampling

Blood sampling was collected between 8.00 and 9.00 a.m. for routine biochemical evaluations, including blood cells count, CRP and vitamin D. The serum level of 25-(OH)-vitamin D were measured using a chemoluminescent immunoassay on a Liaison automatic analyzer (Liaison 25 OH Vitamin D TOTAL Assay, DiaSorin, Saluggia (VC), Italy). Data were expressed as nanograms per milliliter. CRP was evaluated by latex immunoturbid assay (normal value 100–6000 μg/L).

### Statistic Analysis

Statistical analyses were performed using SPSS (Statistical Package of Social Sciences, Chicago, IL, USA) software version 23. Descriptive statistics were performed expressing continuous data as means with SDs and categorical data were expressed by frequency and percentage. Comparisons were evaluated using a t-test, ANOVA test and a chi-square test after assessing normality with the Kolmogorov-Smirnov test. Linear regression analysis was performed to determine the presence of an independent relationship between MPV, vitamin D, PLT and CRP. Moreover an ordinal logistic regression is used to predict the severity of sleep disordered breathing (considering 0 as HC, 1 as PS and 2 as OSAS) given MPV, vitamin D, PLT and CRP. The relationship between these variables was analyzed using Pearson’s correlation. A p-value less than 0.05 was considered statistically significant.

## Results

Children affected by SDB had a mean age of 8.49+2.19 and were prevalently males (23 females, 34%; 44 males, 66%). Descriptive statistics of the samples of PS, OSA and HC is shown in Table 1.

**Table 1:** Descriptive statistics data.

	HC	PS	OSAS	p value
**Number**	70	45	22	/
**Age (years)**	9.04±3.91	9.00±1.75	7.62±3.09	ns
**Females**	30 (43%)	16 (35%)	7 (32%)	ns
**Males**	40 (57%)	29 (65%)	15 (68%)	ns
**Adenoid hypertrophy**	/	26	18	ns
**Tonsillar hypertrophy**	/	28	20	ns
**BMI**	17.56±4.8	18.29±3.60	16.78±.40	ns
**MPV (fL)**	7.57±0.56	8.09±0.94	8.48±0.93	<0.001
**PLT (x10**^**9**^**/L)**	305.03±87.29	291.06±70.59	242.68±54.17	0.01
**CRP (**μ**g/L)**	812.31±456.38	2102.44±5320.08	3242.11±6197.25	0.03
**Vitamin D (ng/mL)**	34.07±11.11	26.21±10.7	20.80±7.57	<0.001
**Mean SpO2**	/	97.3±1.4	90.4±19.6	0.006
**AHI**	/	0.4±0.2	6.5±2.1	<0.001

HC, Healthy Controls; PS, Primary Snoring; OSAS, Obstructive Sleep Apnea Syndrome; BMI, Body Mass Index; MPV, Mean Platelet Volume; PLT, Platelet count; CRP, C Reactive Protein; AHI, Apnea-hypopnea index.

**Fig 1:**
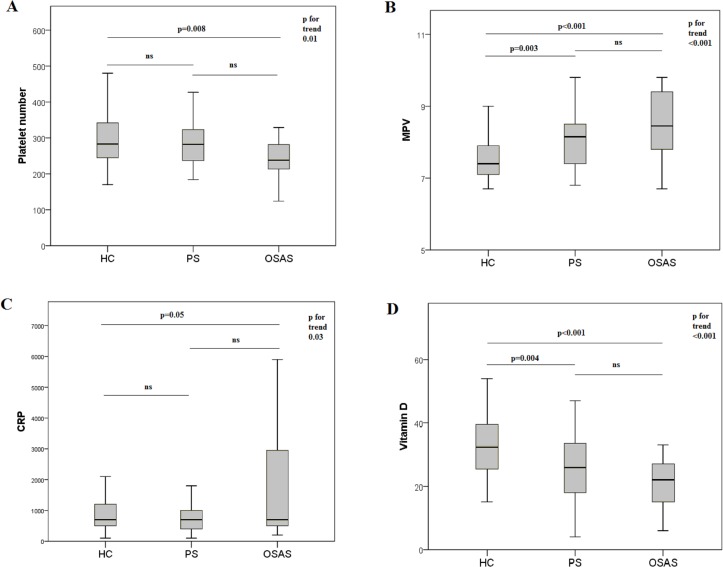
Platelet number, Mean Platelet Volume, C Reactive Protein, and Vitamin D levels in Healthy Controls, primary Snoring and Obstructive Sleep Apnea Syndrome children. (A) Platelet number (x10^9^/L); (B) MPV(fL), Mean Platelet Volume; (C) CRP (μg/L), C Reactive Protein; (D) vitamin D (ng/ml); HC, Healthy Controls; PS, Primary Snoring children; OSAS, Obstructive Sleep Apnea Syndrome children. P values were calculated with Anova and a post hoc Bonferroni test.

MPV was lower in OSAS and PS subjects when compared to HC, although this difference was not significant when comparing specifically OSAS and PS; PLT and CRP levels were higher while vitamin D levels were lower in children with SDB when compared to HC (Table 1 and Fig 1). MPV was correlated with PLT (r = -0.54; p<0.001), vitamin D (r = -0.39, p<0.001), CRP (r = 0.21; p<0.01), SpO2 (r = -0.23; p<0.05) and AHI (r = 0.29; p = 0.009). Moreover vitamin D was related with AHI (r = -0.27; p≤0.01) while vitamin D levels, age and CRP were not significantly related.

A multiple regression was run to predict MPV levels from vitamin D, CRP and PLT. The assumptions of linearity, independence of errors, homoscedasticity, unusual points and normality of residuals were met. These variables statistically significantly predicted MPV, F = 17.42, p<0.0001, adjusted R^2^ = 0.37. Only platelet count and vitamin D added statistically significantly to the prediction, p<0.05. Regression coefficients and standard errors can be found in Table 2.

**Table 2:** Multiple regression coefficients and standard errors.

Variable	B	SE_B_	β
Vitamin D	-0.14	0.006	-0.191 *
PLT	-0.005	0.001	-0.483 *
CRP	0.00002	0.000	0.153

*p<0.05

B, unstandardized regression coefficient; SE_B_, Standard error of the coefficient; β,standardized coefficient; PLT, Platelet count; CRP, C reactive protein.

In children with PS and OSAS another multiple regression was performed to further investigate the effect of SpO2 and AHI on MPV, Vitamin D and PCR and the only significant model showed as independent predictive variable associated with MPV, AHI (SE = 0.37; B = 0.25; p<0.05) with R2 = 0.11.

Moreover the ordinal logistic regression showed an increase in vitamin D and platelet count is associated with a decrease in the odds of having a more severe form of SDB, hence OSAS instead of PS, with an odds ratio of 0.941 (95% CI 0.902, 0.981) Wald χ^2^ = 8.069, p<0.0005 and 0.992 (95% CI 0.985, 0.999) Wald χ^2^ = 4.984 p<0.05 respectively.

MPV, PLT, Vitamin D and CRP values were comparable between children with adeno-tonsillar and without adenoid or tonsillar hypertrophy (data not shown).

## Discussion

The spectrum of SDB is characterized by persistent upper respiratory tract resistance leading to hypoxia, with activation of the sympathetic nervous system and endothelial dysfunction.[24] PS is currently viewed as the minimum anchor point of this spectrum, even though, despite the absence of detectable intermittent hypoxia or repeated arousal, children with PS (70% of children with SDB) experience similar cognitive and behavioral deficits and develop similar oxidative stress mediators compared to children with OSA [21,22,24]

In our sample, we found higher MPV in children with SDB and a correlation between MPV and PLT. Although this result is in line with previously published studies,[11] results in children are often conflicting: while in 2013 Cengiz et al.[17] concluded that children with OSAS caused by chronic tonsillitis and adenoid hypertrophy is associated with low MPV values, in 2014 Onder et al.[18] found no significant relation between MPV and obstructive adenoid hypertrophy while recently Soyaliç et al.[19] reported higher MPV in children with obstructive adenotonsillar hypertrophy when compared to healthy children. The inverse correlation between MPV and PLT has been described as tendency to maintain hemostasis by preserving a constant platelet mass.[30] Another interesting point is that AHI resulted an independent predictive variable associated with MPV. Similar findings were already argued by Varol et al. in 2010 in adults with OSA [31]. Inflammatory process, underlying SDB,[13] may influence thrombopoiesis regulation and, hence, be the origin of decreased platelet volume.[30] The role of MPV as a marker of inflammation, disease activity and efficacy of antinflammatory treatment in several chronic inflammatory disorders is well known, although, in different inflammatory conditions, the direction of its reported changes is often opposite. In this perspective, potentially conflicting results may be explained by the evidence that the size of circulating platelets is dependent on the intensity of systemic inflammation, which can be viewed as a distinctive factor for classifying conditions associated with large and small-sized circulating platelets. High-grade inflammatory conditions (example inflammatory bowel syndrome, rheumatoid arthritis or Familial Mediterranean Fever) are associated with circulation of predominantly small platelets, whereas the same disorders at remission and controlled by antinflammatory drugs are associated with large circulating platelets.[9]

A novel aspect emerging from the present study is that MPV levels are comparable in children with OSAS and with PS as shown in Fig 1. This result underlines the importance of considering Primary Snoring not only as a milder form of OSAS, but rather as a sleep disorder with its own dignity and characteristics, where an undetectable form of hypoxia and many potential factors other than oxygen desaturation might contribute to the inflammation underlying this condition. In this direction, our research group have recently shown, in PS children, higher levels of serum isoprostanes, soluble NOX2 and lower FMD when compared to HC.[24] A possible explanation for this is that primary snorers experience more microarousals (shorter than 3 seconds and sufficient to reestablish airway patency), greater sleep fragmentation, and/or more episodes of subthreshold oxygen desaturation compared with normal controls [32] and that routine PSG might not detect all the alterations by peripheral arterial tonometry [33]. Moreover, although CRP levels did not add a specific singular significant contribution to MPV prediction in the regression analysis, they were progressively higher from HC to OSAS. In 2004 Tauman et al.[13] reported increased CRP levels among children with SDB and a correlation with OAHI, highlighting particularly prominent changes in children who were sleepy or presented neurobehavioral complaints. These authors hypothesized that, in children affected by SDB, the magnitude of inflammatory responses may ultimately lead to cardiovascular, cognitive, and behavioral morbidities. Also Gozal et al. in 2007 [14] reported higher CRP levels in children with OSA developing neurocognitive deficits, and suggested inflammation elicited by OSA as a major determinant of increased risk for neurocognitive dysfunction. Of note these studies were performed on children with a AHI>1 and Abdelnaby Khalyfa in 2011 [34] could not find any significant difference in CRP levels between children with PS and controls. Present results confirm this finding, but, further investigate and report a significant trend in CRP from HC to PS and OSAS. Moreover, results concerning the relation between MPV and CRP should be analyzed considering that adiposity is one of the major determinant of CRP in children [35] and in our sample overweight children were exclude and that a follow up of these children would be interesting to assess the potential increase in CRP over time. Moreover, results should be regarded in the light of the small sample size which may be identified as a limitation of the present study and hence, they should be confirmed by further studies on larger sample of children. Another potential limitation is the lack of polysomnographic data of healthy subjects although these patients did not report any symptom to justify such an expensive and time consuming exam, especially in children.

Furthermore, in the present study, vitamin D levels were lower in PS and OSAS when compared to HC and they were significantly related with MPV; the decrease in vitamin D levels is associated with an increase in the odds of having OSAS instead of PS. The relationship between vitamin D and inflammation is still controversial. [36] One of the main hypothesis is that inflammation may lower 25(OH)D concentration via oxidative stress resulting in the oxidative catabolism of 25(OH)D. [37] We speculate that the interference of an oxidative stress on metabolizing enzymes responsible of liver’s biosynthesis of 25(OH)D and the evidence of oxidative environment in children with PS (24) might be helpful to understand these results. In 2013 Mete et al.[38] showed that lower Vitamin D levels correspond to an increase of OSAS severity independently from BMI values. Although the relation between sleep and vitamin D levels is still controversial, many mechanisms may be implied: for example, TNF-alpha has a role in the pathogenesis of sleep apnea syndrome and is negatively correlated with vitamin D,[39] and the need for sleep during daytime may decrease exposure to sunlight.[40] On the other hand, the relationship between Vitamin D and MPV found in the present study is in line with results shown by Cumhur Cure in 2014 [41] that showed that low vitamin D is independently associated with a high MPV. Raid et al.[42] hypothesized an inverse correlation between tonsils size and vitamin D, although this correlation was not statistically confirmed on multiple linear regression analysis. In the present study, MPV, PLT, Vitamin D and CRP values were comparable between children with adeno-tonsillar and without adenoid or tonsillar hypertrophy. Moreover, vitamin D levels were not related with CRP, partially in line with authors reporting the association between vitamin D and CRP as potentially biased by confounding factors.[43] In this sense the lack of an additional inflammatory marker other than CRP has to be disclosed as a potential limit of this study.

Vitamin D deficiency exerts anticoagulant effects by upregulating the expression of an anticoagulant glycoprotein (thrombomodulin) and downregulating the expression of a critical coagulation factor (tissue factor) in monocytes,[44] and increases itself the release of proinflammatory cytokines such as IL-6 and TNF-α [45] that may lead to a high MPV.[41] Consequently, high MPV, but also vitamin D deficiency per se, increases cardiac disease risk. In adults, Vitamin D deficiency is associated with an increase of cardiovascular diseases, in particular when 25-(OH)-vitamin D level is below 30 ng/mL.[46] Many mechanisms may be implied in this link between vitamin D deficiency and cardiovascular disease, such as the participation of 1,25-(OH)-vitamin D in the regulation of renin-angiotensin axis and in the modulation of smooth muscle cell proliferation, as well as inflammation and thrombosis.

## Conclusions

In conclusion, the present study provides the evidence of higher MPV and vitamin D deficiency in children with PS, as well as in children with OSAS, and assess the potential role of an underlying inflammation, hence, highlighting the importance of an early diagnosis of this previously considered benign form of SDB.
